# Effect of occlusal reduction on post-operative pain of symptomatic and asymptomatic molar teeth

**DOI:** 10.2340/aos.v84.43872

**Published:** 2025-06-11

**Authors:** Aysenur Kamacı Esen, Fatma Furuncuoğlu, Fatima Betul Basturk, Muhammet Nuri Taşcıoğlu, Masoud Parirokh

**Affiliations:** a Department of Endodontics, Sakarya University, Sakarya, Türkiye; b Department of Endodontics, Istanbul Gelisim University, Istanbul, Türkiye; c Department of Endodontics, Kerman University of Medical Sciences, Kerman, Iran

**Keywords:** analgesics, occlusal reduction, post-operative pain, single-visit endodontic treatment, time interval

## Abstract

**Objective:**

This study aimed to compare the intensity of post-operative pain after single-visit root canal treatment of symptomatic or asymptomatic teeth following occlusal reduction.

**Methods:**

A total of 140 symptomatic or asymptomatic patients in need of root canal therapy were registered in this prospective, single-centre, single-blind, randomised clinical trial. For all patients, root canal treatment was carried out in a single visit, and the teeth were restored using composite resin. The patients were randomly allocated into four treatment groups, two of which included occlusal reduction while two treatments left the occlusional contacts intact. Patients’ pain were assessed using a 0–3 verbal rate scale 1, 3, and 7 days following root canal treatment. The pain incidence and intensity were compared using the chi-square and Fisher’s exact tests.

**Results:**

Overall, the post-operative pain intensity was low. Symptomatic individuals had significantly more discomfort at day 1 post-operatively compared to asymptomatic patients (*p* < 0.008). The pain incidence significantly decreased over time for symptomatic patients. When compared to asymptomatic patients without occlusal reduction, symptomatic patients with occlusal reduction had a greater pain incidence at day 3 (*p* < 0.011). For other time intervals, no significant differences in post-operative pain incidence or intensity were found.

**Conclusion:**

Single-visit root canal treatments involving occlusal reduction in both symptomatic or asymptomatic molars had no significant effect on post-operative pain.

## Introduction

Post-operative pain is a type of discomfort that develops following root canal therapy. Post-operative pain is uncomfortable for both patients and clinicians, and it begins a few hours after endodontic treatment [1]. Studies indicate that a significant number of patients experience post-operative pain following endodontic therapy [2–4]. The incidence of post-operative endodontic pain has been reported in 24% of cases on average, but it may increase up to 80% within the first 24 hours [5–7].

Several factors can contribute to the development of post-operative pain including pre-operative discomfort, traumatic occlusion, root canal obturation technique, tooth type, age, gender, and the presence of pre-operative pain [8]. As a result, managing discomfort during root canal therapy is a crucial concern. Multiple clinical investigations have evaluated the impact of various factors on post-operative pain, including the use of intracanal medication, single- or multiple-visit endodontic therapy, and the impact of irrigation activation [1, 9–11].

One key factor linked to post-operative discomfort is the effect of occlusal reduction. This has been a significant focus of research, because pain following endodontic treatment remains a major concern for both patients and practitioners. Studies have shown that occlusal reduction can help alleviate pain by minimising mechanical allodynia and reducing the activation of hypersensitive nociceptors [12]. Extensive research has been conducted to evaluate its impact both before and after endodontic treatment.

However, despite extensive research on the topic, the effect of occlusal reduction on post-operative pain remains controversial. While some studies suggest that occlusal reduction helps alleviate discomfort following endodontic treatment [13–15], others find no significant benefit in the management of post-operative pain [16–18]. These conflicting results may be due to differences in case selection criteria and treatment approaches across studies. Given these variations, the current study aims to evaluate the impact of occlusal reduction on post-operative pain in both symptomatic and asymptomatic molar teeth.

The study was designed using the Population, Intervention, Comparison, Outcome, and Study [4, 19] framework to investigate whether occlusal reduction (I) reduces post-operative pain (O) compared to teeth without occlusal reduction (C) in symptomatic and asymptomatic patients (P) following single-visit root canal treatment in a randomised clinical trial (S). The null hypothesis states that occlusal reduction has no significant effect on post-operative pain after single-visit root canal treatment.

## Materials and methods

A prospective, single-centre, single-blind, randomised clinical trial was designed with the approval of the Sakarya University Faculty of Medicine Ethical Committee (Approval number: E-71522473-050.01.04-39802-329), and the research protocol was retrospectively registered in the ClinicalTrials.gov database (NCT/06953856). Volunteers who accepted to participate in this study were informed about the procedure. A written consent was signed, and a copy was given to volunteers.

### Sample size calculation

Minimum sample size was determined as 30 [20] using G*Power 3.1 (Heinrich Heine University, Düsseldorf, Germany) software from a similar study. This minimum value served as the basis for determining the number of samples, as per the groups.

### Patient selection and allocation

A total of 325 patients with a non-contributory medical history applied to Sakarya University Faculty of Dentistry Department of Endodontics with endodontic treatment needs between August 2020 and January 2021.

**Figure 1:**
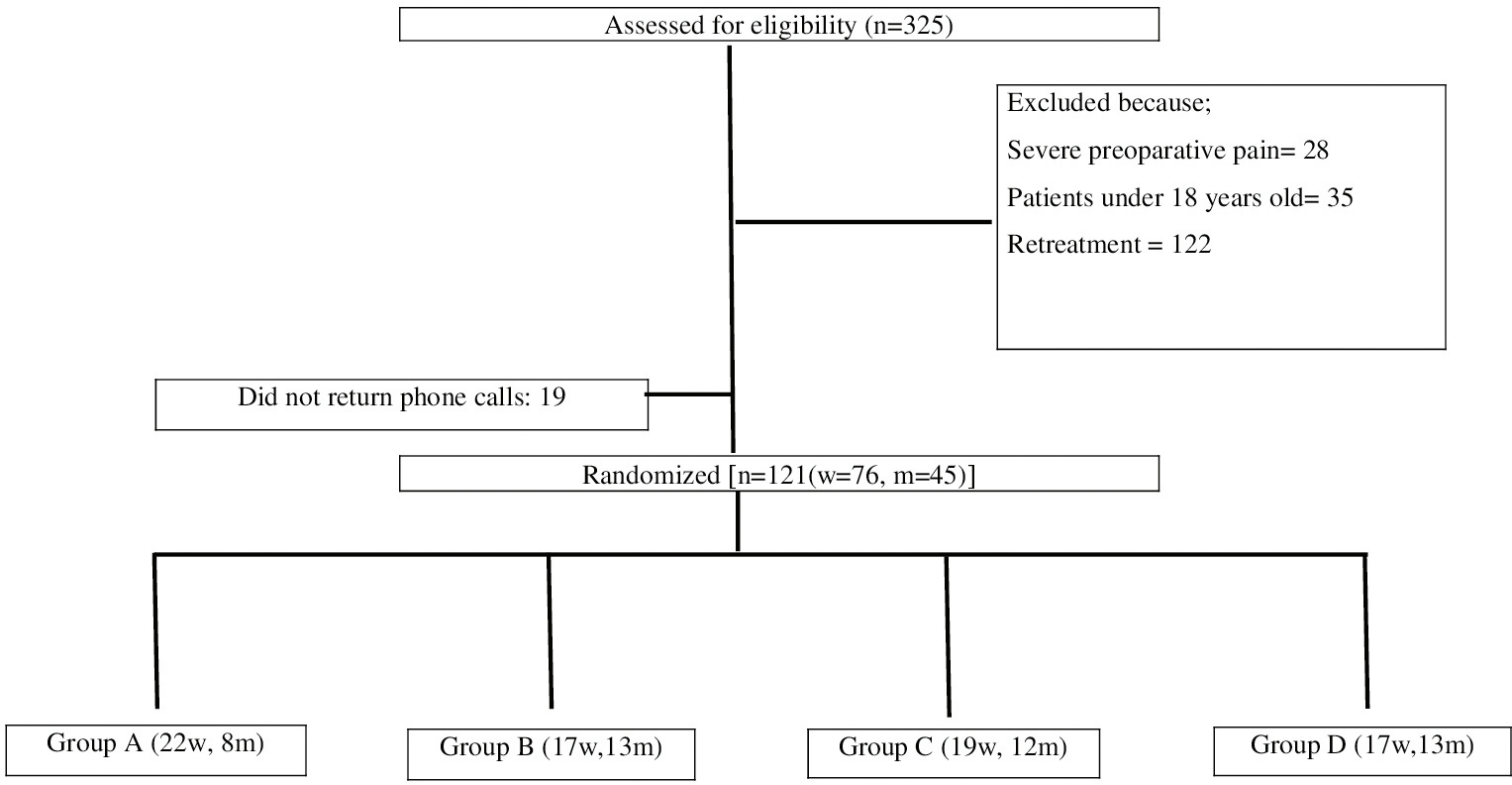
Flowchart of patients’ distribution to the groups.

The inclusion and exclusion are listed in Table 1. The clinical diagnosis of irreversible pulpitis was confirmed through periapical radiographs, patient dental history, and a combination of diagnostic tests, including the electric pulp test (Parkell Inc., Farmingdale, NY, USA) and the cold test (Roeko Endo-Frost; Roeko, Langenau, Germany), particularly in cases where pain assessment was unclear. In addition, the duration of pulp bleeding during root canal therapy was also taken into account when diagnosing irreversible pulpitis. Figure 1 shows the patients’ distribution to the groups.

**Table 1:** Criteria for inclusion and exclusion of patients.

Inclusion criteria	Exclusion criteria
Healthy patients	Patients under 18 years old
Maxillary and mandibulary first and second molars	Patients older than 65 years old
Irreversible pulpitis case with or without prior discomfort	Teeth that have previously undergone root canal treatment
	Complicating systemic diseases
	Allergies to local anesthetic agents
	Presence of acute apical abscesses
	History of trauma
	Analgesic, anti-inflammatory, or antibiotic intake during the 7 days before treatment
	Periodontal pockets deeper than 4 mm
	Multiple teeth that require endodontic treatment
	Pregnancy
	Teeth that cannot be restored
	Patients who needed emergency treatment
	Grade 2 or 3 mobility
	Patients with bruxism

A total of 140 patients fulfilled the inclusion criteria and were selected to take part in this clinical trial. Informed consent was obtained from all patients after they were fully informed about the procedure. The patients were notified that they would be contacted on the first, third, and seventh days after treatment, and they were given the option to reach out in case of any unexpected issues or severe pain.

### Randomisation

Due to ethical considerations, patients requiring full cuspal coverage restorations who were referred from the prosthodontics department were randomly allocated to the treatment groups. All intervention and control treatment groups were assigned a number and masked from the patients. The patients scheduled to receive crowns after root canal therapy were asked to select an envelope containing a number that assigned them to one of the four treatment groups.

### Root canal treatment procedure

The same endodontist (AKE), with a minimum of 5-year experience performed all root canal treatments in a single visit. The groups were as follows (*n* = 35):

Group A: Symptomatic teeth, occlusal contacts left intact

Group B: Asymptomatic teeth, occlusal contacts left intact

Group C: Symptomatic teeth that underwent occlusal reduction

Group D: Asymptomatic teeth that underwent occlusal reduction

Local anaesthesia was administered using 3.6 mL of lidocaine with 1:100,000 epinephrine (Adeka İlaç A.Ş, Turkey), and the affected tooth was isolated with a rubber dam. An access cavity was prepared, and any existing coronal restorations were removed. The root canal length was determined using a size #10 K-file (Micro-Mega, Besançon, France) in conjunction with an electronic apex locator (Woodpex-III, Guilin Woodpecker Medical Instrument Co., Ltd., China), with measurements taken until the device indicated a 0.0 reading. The working lengths were then calculated by subtracting 1 mm from that point. Depending on the condition of the individual root canal, apical preparation was performed using ProTaper Next rotary instruments (Dentsply Maillefer, Ballaigues, Switzerland), selecting two sizes larger than the initial apical binding file.

Between each instrument, root canals were irrigated with 2 mL of 3% NaOCl (Coltene/Whaledent, Switzerland) with a 30G side-vented endodontic needle (NaviTip, Ultradent, UT, USA) positioned 2 mm short from the working length. Before obturation, x-rays were taken with matched Gutta Percha, and final irrigation was performed as follows: 2 mL of 5% EDTA, 4 mL of 3% NaOCl, and 2 mL of distilled water.

All treatments were performed in a single session, and all teeth were obturated with AH Plus root canal sealer (Dentsply Maillefer, Ballaigues, Switzerland) and matched Gutta Percha cones. All occlusal reduction cases were selected from teeth in need of full cuspal coverage restorations. Restoration of the teeth was done in the same session using bulk-fill resin (SDR, Dentsply/DeTrey, Konstanz, Germany) and posterior resin composite (Estelite Posterior Quick, Tokuyama, Tokyo, Japan).

All occlusal contacts in the intervention groups (groups C and D) were removed and those in the control groups (groups A and B) were maintained.

The patients were advised to take 200 mg of ibuprofen every 8 hours as needed for pain management. Following the procedure, they were contacted on the first, third, and seventh days to assess and document their pain levels using the Verbal Rating Scale (VRS). The scale ranges from 0 to 3, where 0 indicates no discomfort or pain, 1 represents mild pain (not requiring analgesic medication), 2 signifies moderate pain (requiring analgesic medication), and 3 denotes severe pain (interfering with physical activity and minimally responsive to analgesics).

### Statistical analysis

The data were analysed using IBM SPSS version 26.0 (SPSS Inc., Chicago, IL, USA). The Chi-square test was used to compare categorical variables between groups. The daily pain distributions by groups were assessed using the Friedman and Fisher’s Exact tests.

## Results

Out of 325 patients, 140 were enrolled in the study (Figure 1). However, 19 patients were excluded due to failure to respond to follow-up phone calls regarding pain assessment. The remaining 121 patients (40 male and 81 female) completed the study, with 61 in the intervention and 60 in the control groups. In the intervention group, there were 25 males and 36 females, whereas in the control group, there were 21 males and 39 females. In addition, 18 smokers were in the intervention group, compared to 7 smokers in the control group. There was no statistically significant difference between the groups in terms of gender distribution (*p* = 0.5) or smoking status (*p* = 0.115) (Table 2). Pre-operative pain was reported by 60 patients, with half of these individuals belonging to the intervention group.

**Table 2:** Comparisons of categorical variables by groups.

	Group A	Group B	Group C	Group D	Total	*P*
Smoking habit						
No	21 (70%)	21 (70%)	27 (87.1%)	25 (89.3%)	94 (79%)	0.115
Yes	9 (30%)	9 (30%)	4 (12.9%)	3 (10.7%)	25 (21%)	
Gender						
Male	8 (26.7%)	13 (43.3%)	12 (38.7%)	13 (43.3%)	46 (38%)	0.500
Female	22 (73.3%)	17 (56.7%)	19 (61.3%)	17 (56.7%)	75 (62%)	
Analgesic intake						
No	23 (76.7%)	26 (86.7%)	22 (71%)	27 (90%)	98 (81%)	0.204
Yes	7 (23.3%)	4 (13.3%)	9 (29%)	3 (10%)	23 (19%)	

**Figure 2:**
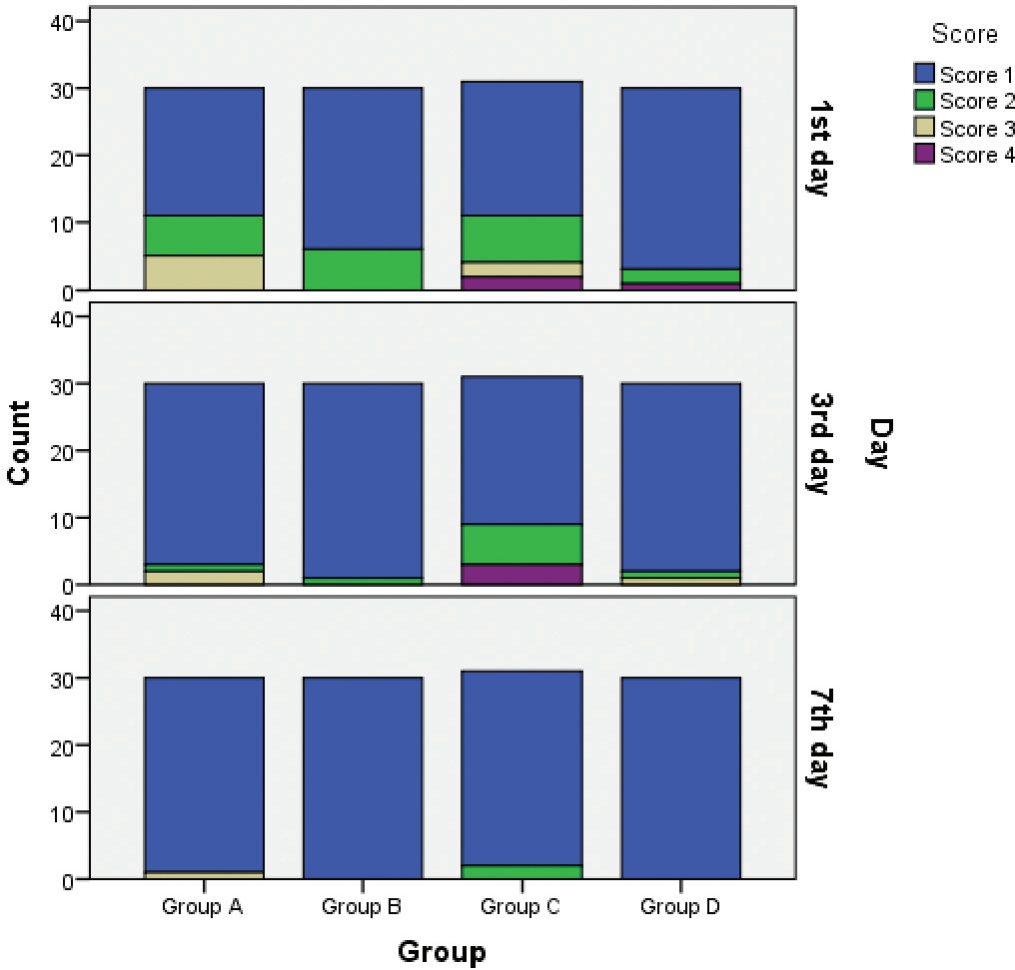
The pain distribution by group on the first, third, and seventh days.

The mean pain scores are presented in Figure 2. They indicate that asymptomatic patients experienced significantly less pain than symptomatic patients on post-operative day 1 and day 3. On post-operative day 1, the pain intensity was significantly higher in symptomatic patients (groups A and C) compared to asymptomatic (groups B and D) patients (*p* < 0.008). However, the pain incidence gradually decreased over time in groups A and C. By day 3, the symptomatic patients continued to report significantly more discomfort than the asymptomatic patients (*p* < 0.011). By day 7, there were no significant differences in post-operative pain incidence or intensity between the groups.

The pain incidence and intensity gradually decreased over time in all groups, with no significant difference between them (*p* > 0.05). By post-operative day 7, there were no significant differences in post-endodontic pain among the groups (*p* > 0.05). However, on day 3, the differences between groups C and D and groups A and B were statistically significant (*p* < 0.011). Overall, occlusal reduction in molar teeth had no significant impact on post-operative pain following single-visit root canal treatment (*p* > 0.05).

## Discussion

This study found that occlusal reduction had no significant effect on post-operative pain following single-visit root canal treatment in both symptomatic and asymptomatic molar teeth. The pain incidence and intensity were higher in both groups during the first 3 post-operative days but significantly decreased by day 7.

Several methods have been used to assess post-operative endodontic pain, often comparing pain intensity at different time points using the Visual Analogue Scale (VAS) [21] or multi-level pain classification systems [16]. We used a four-level VRS, selected for its simplicity for patients to use. Moreover, previous studies have reported that the pain classification system had no effect on the post-operative pain results across different time intervals [21, 22].

This study is a randomised, single-blind, single-centred clinical trial with patient-reported outcome measures. Participants were blinded to the interventions used to minimise performance and detection bias.

All root canal treatments in this study were performed in a single visit, as systematic reviews and meta-analyses did not reveal any significant difference in the incidence or the intensity of post-operative pain between single-visit and multiple-visit root canal treatment, even in necrotic teeth [23, 24].

Recent systematic reviews have highlighted the need for further clinical trials to better understand the role of occlusal reduction in post-operative pain [5]. However, few studies have investigated the effect of occlusal reduction in single-visit cases with a wider range of pulpal and periapical conditions. Most previous research has focused on patients with symptomatic irreversible pulpitis [25] with tenderness to percussion [15, 16] or with non-vital teeth [4]. This study, however, included vital teeth with or without symptoms and randomly allocated patients to two balanced groups based on the intervention.

Several confounding factors can influence post-endodontic pain, including gender, tooth type, and pre-operative pain [4, 13]. To minimise these effects, the study balanced the intervention and control groups based on each confounder; the gender, pre-operative pain, and smoking habit. There was no statistically significant difference in the incidence or intensity of post-operative pain between groups based on gender.

Previous studies on the effect of occlusal reduction have included both molars and premolars [13, 16], whereas some focused solely on mandibular molars [4, 22]. In this study, both maxillary and mandibular first and second molars were included to provide a broader evaluation of tooth type.

The cause of post-operative pain might be attributed to debris extrusion, instrument kinematics, and apical third enlargement [12]. Given the variation in initial apical diameters across each root canal, we decided to standardise the apical preparation to two sizes larger than the initial apical binding file with a 6% or 7% taper. A previous study comparing different preparation sizes and tapers found that apical enlargement to two sizes larger than the initial apical file with a 4% taper had lower success rates compared to a 6% taper [26]. Since all instrumentation techniques cause some degree of apical debris extrusion, this can trigger an inflammatory response in periradicular tissues [12]. In a recent systematic review and meta-analysis [27], rotary instrumentation resulted in a lesser debris extrusion into the periapical areas compared to reciprocation; therefore, in the present study, each tooth was treated using rotary instruments with a crown-down approach to minimise debris extrusion.

After chemo-mechanical preparation, the same operator completed the root canal treatment. The amount of apically extruded obturation material might differ according to the filling techniques. Warm vertical compaction technique had a higher percentage of sealer extrusion to periradicular tissues [23]. In the studies where filling techniques were the same between groups, the choice of sealer did not affect post-operative pain [28]. Therefore, the root canals were filled using the lateral compaction technique with a resin-based sealer. One of the limitations of this study is the lack of contribution of several operators, which might have enhanced the external validity of the results [4, 29].

All occlusal reduction cases in this study were selected from teeth requiring full cuspal coverage restorations, allowing for the inclusion of asymptomatic patients as well. However, if a tooth undergoing occlusal reduction does not receive a full cuspal coverage afterwards, it may lose functionality [16]. Thus, clinicians should inform patient about the potential drawbacks of occlusal reduction [16].

In addition, the presence of pre-operative pain might be a strong predictor of post-operative pain [7, 16], a finding that was confirmed in this study. On post-operative days 1 and 3, the symptomatic patients reported higher levels of pain incidence and intensity compared to the asymptomatic patients. However, by day 7, there was no statistically significant difference in post-operative pain between the two groups.

In the present study, patients were categorised based on the presence or absence of pre-operative pain, with the primary objective being to assess pain resolution following occlusal reduction rather than comparing the severity of the pre-operative pain. Consequently, only the presence or absence of pain was considered in the analysis. However, given the potential consequences of variations in pre-operative pain levels among groups, this circumstance may be regarded as a limitation of the present study.

Clinical trials are required to be uploaded prospectively to a clinical trial database. However, this study was uploaded retrospectively, despite the initial intention for prospective submission. This deviation may be considered as another limitation of the current study, as it could potentially increase the risk of bias.

The results revealed that pain incidence and intensity were most evident during the first three post-operative days, significantly decreasing thereafter. By day 7, over 96% of patients reported an absence of any level of discomfort, supporting a previous research by Ahmed et al. [15] suggesting that pain reduction may be attributed to the healing of a symptomatic pulp after undergoing pulpectomy. This study confirms that even in symptomatic teeth, substantial periapical healing occurs by the seventh day following root canal therapy.

## Conclusion

Occlusal reduction in both symptomatic or asymptomatic molars had no significant impact on post-operative pain following single-visit root canal treatment. However, it is important to note that occlusal reduction is not an independent determinant for the relief of post-endodontic pain.
